# Supplementary material for: BDH1 acetylation at K116 modulates milk fat production in dairy goats
Source: J Anim Sci Biotechnol. 2025 Dec 22;16:177. doi: 10.1186/s40104-025-01315-5 (PMC12720437; doi:10.1186/s40104-025-01315-5)
Supplement: Supplementary file 1 — Additional file1: Table S1. Design of primers for the BDH1 acetylation mutation site. Table S2. Primers for RT-qPCR analysis. Table S3. Differential acetylation protein and site significance (P-value). [file 40104_2025_1315_MOESM1_ESM.pdf]

Supplementary material

Supplementary Table

Table S1 Design of primers for the BDH1 acetylation mutation site

Target genes	Primer sequences (5' to 3')
pcDNA3.1-5×Flag-BDH1-K91R -F	AATGAAGGACAGAGGGAGTGA
pcDNA3.1-5×Flag-BDH1-K91R -R	TCACTCCCTCTGTCCTTCATT
pcDNA3.1-5×Flag-BDH1-K91Q -F	AATGAAGGACCAAGGGAGTGA
pcDNA3.1-5×Flag-BDH1-K91Q -R	TCACTCCCTTGGTCCTTCATT
pcDNA3.1-5×Flag-BDH1-K116R -F	AACGTCTGCAGAAGTGAAGA
pcDNA3.1-5×Flag-BDH1-K116R -R	TCTTCACTTCTGCAGACGTT
pcDNA3.1-5×Flag-BDH1-K116Q -F	AACGTCTGCCAAAGTGAAGA
pcDNA3.1-5×Flag-BDH1-K116Q -R	TCTTCACTTTGGCAGACGTT
pcDNA3.1-5×Flag-BDH1-K122R -F	GAGGTGGATAGAGCAGCAGAG
pcDNA3.1-5×Flag-BDH1-K122R -R	CTCTGCTGCTCTATCCACCTC
pcDNA3.1-5×Flag-BDH1-K122R -F	GAGGTGGATCAAGCAGCAGAG
pcDNA3.1-5×Flag-BDH1-K122R -R	CTCTGCTGCTTGATCCACCTC

Table S2 Primers for RT-qPCR analysis

Target genes	Gene bank	Primer sequences (5' to 3')	PCR product size, bp
BDH1	XM_018046468.1	F:AGGCTCGCTGCTGTTTAACT	201
		R:CCATCACTCCCTTTGTCCTTCA	
SCD1	NM_001285619.1	F:CCATCGCCTGTGGAGTCAC	256
		R:GTCGGATAAATCTAGCGTAGCA	
SREBP1	NM_001285619.1	F:CTGCTGACCGACATAGAAGACAT	81
		R:GTAGGGCGGGTCAAACAGG	
LXRα	NM_001285619.1	F:CATCAACCCCATCTTCGAGTT	163
		R:CAGGGCCTCCACATATGTGT	
FASN	NM_001285629.1	F:GGGCTCCACCACCGTGTTCCA	226
		R:GCTCTGCTGGGCCTGCAGCTG	
ACCα	XM_018064168	F: CTCCAACCTCAACCACTACGG	171
		R:GGGGAATCACAGAAGCAGCC	
ELOVL6	NM_001314257.1	F: GGAAGCCTTTAGTGCTCTGGTC	205
		R:ATTGTATCTCCTAGTTCGGGTGC	
LPL	NM_001285607.2	F: CATCAACCCCATCTTCGAGTT	163
		R:CAGGGCCTCCACATATGTGT	
FABP3	NM_001285701.1	F:GATGAGACCACGGCAGATG	120
		R:GTCAACTATTTCCCGCACAAG	
ACSL1	NM_001314257.1	F:GTGGGCTCCTTTGAAGAACTGT	120
		R:ATAGATGCCTTTGACCTGTTCAAAT	
CD36	XM_018046617	F:GTACAGATGCAGCCTCATTTCC	81

		R:TGGACCTGCAAATATCAGAGGA	
GPAM	XM_005698479.3	F:ATTGACCCTTGGCACGATAG	188
		R:AACAGCACCTTCCCACAAAG	
XDH	XM_005698479.3	F:GATCATCCACTTTTCTGCCAATG	100
		R:CCTCGTCTTGGTGCTTCCAA	
PLIN1	XM_018066568.1	F:GATGAGACCACGGCAGATG	120
		R:GTCAACTATTTCCCGCACAAG	
UXT	XM_005700842.2	F:TGTGGCCCTTGGATATGGTT	101
		R:GGTTGTCGCTGAGCTCTGTG	

Table S3 Differential acetylation protein and site significance (*P*-value)

Protein accession	Position(K)	Gene name	M/D *P*-value	Involved in access
XP_013823264.1	273	PREB	0.01197338	Lactation
XP_005684485.1	179	EEF1A1	2.98699E-05	Lactation
XP_005684485.1	395	EEF1A1	0.003385246	Lactation
XP_005684485.1	408	EEF1A1	0.006186979	Lactation
XP_005684485.1	244	EEF1A1	0.011841941	Lactation
XP_005684485.1	392	EEF1A1	6.38736E-05	Lactation
XP_005684485.1	439	EEF1A1	0.00066928	Lactation
XP_005684485.1	41	EEF1A1	0.000749596	Lactation
XP_005699833.1	437	EEF1G	1.19034E-05	Lactation
XP_005699833.1	243	EEF1G	0.00673262	Lactation
XP_005699833.1	147	EEF1G	0.000132104	Lactation
XP_005699833.1	404	EEF1G	0.002806335	Lactation
XP_005699833.1	132	EEF1G	0.000225854	Lactation
XP_005682629.2	619	EEF2	0.009323451	Lactation
XP_005682629.2	571	EEF2	0.013830336	Lactation
XP_005682629.2	275	EEF2	0.000173492	Lactation
XP_005682629.2	283	EEF2	0.000119448	Lactation
XP_005682629.2	252	EEF2	0.001613604	Lactation
XP_005682629.2	258	EEF2	3.4135E-05	Lactation
XP_005682629.2	328	EEF2	0.01670385	Lactation
XP_005682629.2	308	EEF2	0.000667169	Lactation
XP_005682629.2	272	EEF2	0.00025468	Lactation
XP_017896097.1	513	EEF2K	0.003391496	Lactation
XP_005691413.1	249	EIF2B1	0.000132867	Lactation
XP_005701053.1	426	EIF2S3	5.51282E-05	Lactation
XP_005701053.1	449	EIF2S3	0.007512501	Lactation
XP_005701053.1	421	EIF2S3	0.00087771	Lactation
XP_017896105.1	187	EIF3B	1.44958E-06	Lactation
XP_017896105.1	183	EIF3B	0.000929544	Lactation
XP_005689215.1	275	EIF3E	0.001774777	Lactation
XP_005689215.1	409	EIF3E	7.81493E-06	Lactation

XP_017913858.1	306	EIF3H	0.001809939	Lactation
XP_013819789.1	207	EIF3L	0.025080557	Lactation
XP_013819789.1	549	EIF3L	9.84508E-05	Lactation
XP_013819789.1	301	EIF3L	0.002227765	Lactation
XP_017914774.1	176	EIF3M	0.001088469	Lactation
XP_013818053.1	422	AGL	0.003025647	Lactose
XP_017894444.1	67	GMDS	0.000108158	Lactose
XP_017897815.1	191	HK1	0.000298309	Lactose
XP_017897815.1	763	HK1	2.80788E-05	Lactose
XP_017910465.1	876	HK2	0.000478381	Lactose
XP_017900590.1	234	PGM1	0.004172825	Lactose
XP_017904854.1	227	PGM2	4.06906E-05	Lactose
XP_017904854.1	496	PGM2	0.000981904	Lactose
XP_017904854.1	501	PGM2	0.006180069	Lactose
XP_017904854.1	60	PGM2	0.001656713	Lactose
XP_017904854.1	558	PGM2	3.78553E-05	Lactose
XP_017904854.1	578	PGM2	0.01187091	Lactose
XP_005700658.1	146	PGK1	0.000938202	Lactose
XP_005700658.1	139	PGK1	0.010069847	Lactose
XP_005700658.1	131	PGK1	6.15368E-06	Lactose
XP_005700658.1	353	PGK1	4.2862E-05	Lactose
XP_005700658.1	11	PGK1	3.37895E-05	Lactose
XP_005700658.1	322	PGK1	0.000597445	Lactose
XP_005700658.1	220	PGK1	0.024503193	Lactose
XP_005700658.1	30	PGK1	0.001753967	Lactose
XP_005696307.1	91	RPL10A	0.000290136	Lactose
XP_005696307.1	130	RPL10A	0.001003019	Lactose
XP_005696307.1	106	RPL10A	0.000630958	Lactose
XP_005696307.1	156	RPL10A	0.000235902	Lactose
XP_005696307.1	212	RPL10A	0.017457283	Lactose
XP_005696307.1	152	RPL10A	0.005898417	Lactose
XP_005676921.1	85	RPL11	0.000128271	Lactose
XP_005676921.1	144	RPL11	0.008553763	Lactose
XP_005676921.1	154	RPL11	0.012917424	Lactose
XP_017922594.1	85	RPL14	0.001176706	Lactose
XP_005690810.1	69	RPL22	0.032122702	Lactose
XP_017920649.1	27	RPL27	0.00122125	Lactose
XP_017920649.1	3	RPL27	0.001051307	Lactose
XP_017920649.1	128	RPL27	0.002606189	Lactose
XP_017920649.1	93	RPL27	5.33028E-05	Lactose
XP_013826895.2	385	BCAT2	0.002519202	Milk Protein
XP_013826895.2	378	BCAT2	8.51682E-06	Milk Protein
XP_013826895.2	322	BCAT2	0.008316104	Milk Protein
XP_013826895.2	48	BCAT2	0.000839365	Milk Protein

XP_013826895.2	247	BCAT2	0.00028481	Milk Protein
XP_013826895.2	74	BCAT2	0.00246399	Milk Protein
NP_001272677.1	1218	MTOR	0.023593361	Milk Protein
XP_005684978.2	305	ARMT1	0.000248033	Milk Protein
XP_005683859.2	116	PSAT1	2.58767E-05	Milk Protein
XP_005683859.2	318	PSAT1	0.006225199	Milk Protein
XP_005683859.2	190	PSAT1	0.008412622	Milk Protein
XP_005697810.1	172	PSPH	5.10049E-05	Milk Protein
XP_017911437.1	295	PTPA	0.004543606	Milk Protein
XP_017911437.1	289	PTPA	0.000102593	Milk Protein
XP_005696027.1	309	QARS1	0.003900127	Milk Protein
XP_005696027.1	673	QARS1	0.00052819	Milk Protein
XP_005696027.1	412	QARS1	0.000710101	Milk Protein
XP_005696027.1	628	QARS1	0.000170533	Milk Protein
XP_005698373.1	99	GOT1	0.000787259	Milk Protein
XP_005692154.2	88	GOT2	4.83643E-05	Milk Protein
XP_005692154.2	120	GOT2	0.000326897	Milk Protein
XP_005692154.2	300	GOT2	0.000170186	Milk Protein
XP_005692154.2	71	GOT2	3.68261E-05	Milk Protein
XP_005692154.2	157	GOT2	8.02161E-05	Milk Protein
XP_005692154.2	402	GOT2	0.002030412	Milk Protein
XP_005692154.2	232	GOT2	5.74642E-05	Milk Protein
XP_005692154.2	80	GOT2	1.06505E-05	Milk Protein
XP_005692154.2	336	GOT2	4.71989E-06	Milk Protein
XP_005692154.2	343	GOT2	6.22143E-05	Milk Protein
XP_005692154.2	394	GOT2	0.000325261	Milk Protein
XP_005692154.2	307	GOT2	0.000327312	Milk Protein
XP_005692154.2	361	GOT2	0.000153569	Milk Protein
XP_005692154.2	183	GOT2	3.10497E-06	Milk Protein
XP_005700926.1	56	HSD17B10	8.58084E-06	Milk Protein
XP_005700926.1	69	HSD17B10	0.000105896	Milk Protein
XP_005700926.1	105	HSD17B10	0.004260598	Milk Protein
XP_005700926.1	52	HSD17B10	1.23501E-07	Milk Protein
XP_005700926.1	99	HSD17B10	0.001762942	Milk Protein
XP_005682987.2	272	HSPA4	0.005027267	Milk Protein
XP_005687195.1	327	HSPA5	0.018727246	Milk Protein
XP_005687195.1	119	HSPA5	0.045473214	Milk Protein
XP_005687195.1	354	HSPA5	0.047412742	Milk Protein
XP_005687195.1	448	HSPA5	0.007253897	Milk Protein
XP_005687195.1	124	HSPA5	0.000704371	Milk Protein
XP_005687195.1	524	HSPA5	0.010846356	Milk Protein
XP_005687195.1	447	HSPA5	0.046842992	Milk Protein
XP_005683052.1	138	HSPA9	0.000492186	Milk Protein
XP_005683052.1	187	HSPA9	0.041393097	Milk Protein

XP_005683052.1	300	HSPA9	0.001022286	Milk Protein
XP_005683052.1	288	HSPA9	8.8277E-05	Milk Protein
XP_005683052.1	345	HSPA9	0.013421246	Milk Protein
XP_005683052.1	612	HSPA9	2.45065E-05	Milk Protein
XP_005683052.1	600	HSPA9	0.000125752	Milk Protein
XP_005683052.1	394	HSPA9	0.000770675	Milk Protein
XP_005683052.1	135	HSPA9	0.000526916	Milk Protein
XP_005677570.1	44	S100A8	0.002322332	Milk Protein
XP_005677570.1	88	S100A8	0.008766551	Milk Protein
XP_005686123.3	169	COQ6	4.05611E-05	Milk Protein
XP_017914891.1	211	ACAD8	0.000110229	Milk Protein
XP_017914891.1	251	ACAD8	0.000347978	Milk Protein
XP_017901373.1	246	AHCYL1	0.000972426	Milk Protein
NP_001301241.1	198	MFGE8	5.2639E-05	Milk Fat
XP_017912771.1	389	ACSS1	6.77923E-05	Milk Fat
XP_017912771.1	219	ACSS1	0.000920484	Milk Fat
XP_017913867.1	82	FABP5	0.001142441	Milk Fat
XP_017913867.1	40	FABP5	0.001057507	Milk Fat
XP_017913867.1	55	FABP5	0.006904573	Milk Fat
XP_017901957.1	122	BDH1	0.002386018	Milk Fat
XP_017901957.1	116	BDH1	0.000166358	Milk Fat
XP_017901957.1	91	BDH1	8.50E-03	Milk Fat
XP_017920606.1	71	STAT5B	0.017537488	Milk Fat
XP_017920606.1	343	STAT5B	0.002017345	Milk Fat
XP_017920606.1	347	STAT5B	0.00066682	Milk Fat
XP_013830723.2	779	GPAM	0.000276328	Milk Fat
XP_013830723.2	775	GPAM	0.002089243	Milk Fat
XP_013827059.1	59	FABP3	1.2576E-06	Milk Fat
XP_013827854.1	383	STAT3	0.000863723	Milk Fat
XP_013827854.1	370	STAT3	0.000863723	Milk Fat
XP_017901474.1	58	PHGDH	0.001347206	Milk Fat
XP_013819152.1	87	ACSS3	5.27546E-05	Milk Fat
XP_013819152.1	415	ACSS3	0.000974908	Milk Fat
XP_013819152.1	214	ACSS3	5.56117E-06	Milk Fat
XP_013819152.1	247	ACSS3	0.000238182	Milk Fat
XP_013819152.1	250	ACSS3	0.000277437	Milk Fat
XP_013819152.1	230	ACSS3	0.018957294	Milk Fat
XP_013819152.1	520	ACSS3	8.32158E-05	Milk Fat
XP_013819152.1	658	ACSS3	0.000269353	Milk Fat
XP_013820554.1	353	GCDH	5.13926E-05	Milk Fat
XP_013820554.1	222	GCDH	0.000126871	Milk Fat
XP_013820554.1	310	GCDH	0.000155862	Milk Fat
XP_013828239.1	462	OXCT1	1.41268E-05	Milk Fat
XP_013828239.1	402	OXCT1	1.41268E-05	Milk Fat

XP_013828239.1	277	OXCT1	1.41268E-05	Milk Fat
XP_013828239.1	157	OXCT1	1.41268E-05	Milk Fat
XP_005686992.1	396	HADHA	0.000317891	Milk Fat
XP_005686992.1	391	HADHA	0.000391072	Milk Fat
XP_005686992.1	554	HADHA	2.62026E-06	Milk Fat
XP_005686992.1	338	HADHA	8.17197E-07	Milk Fat
XP_005686992.1	151	HADHA	0.001278861	Milk Fat
XP_005686992.1	114	HADHA	4.64443E-05	Milk Fat
XP_005686992.1	744	HADHA	0.000440119	Milk Fat
