# Supplementary material for: BDH1 acetylation at K116 modulates milk fat production in dairy goats
Source: J Anim Sci Biotechnol. 2025 Dec 22;16:177. doi: 10.1186/s40104-025-01315-5 (PMC12720437; doi:10.1186/s40104-025-01315-5)
Supplement: Supplementary file 2 — Additional file 2: The original gel and blot images. [file 40104_2025_1315_MOESM2_ESM.pdf]

Figure1B

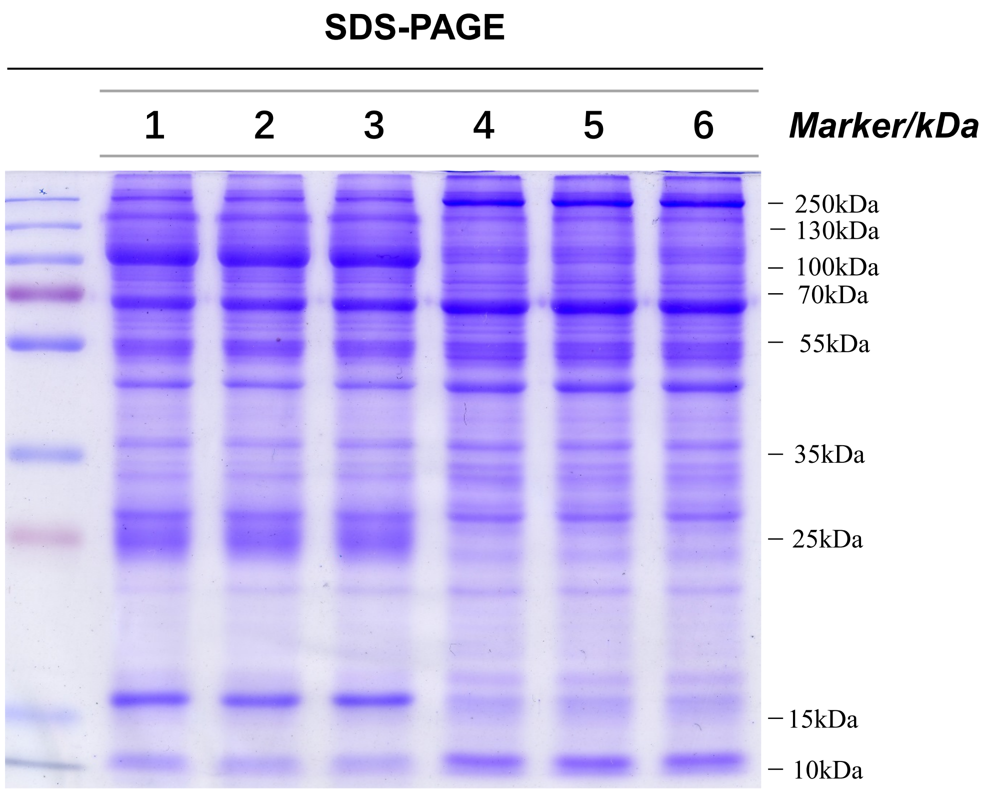

Figure1C

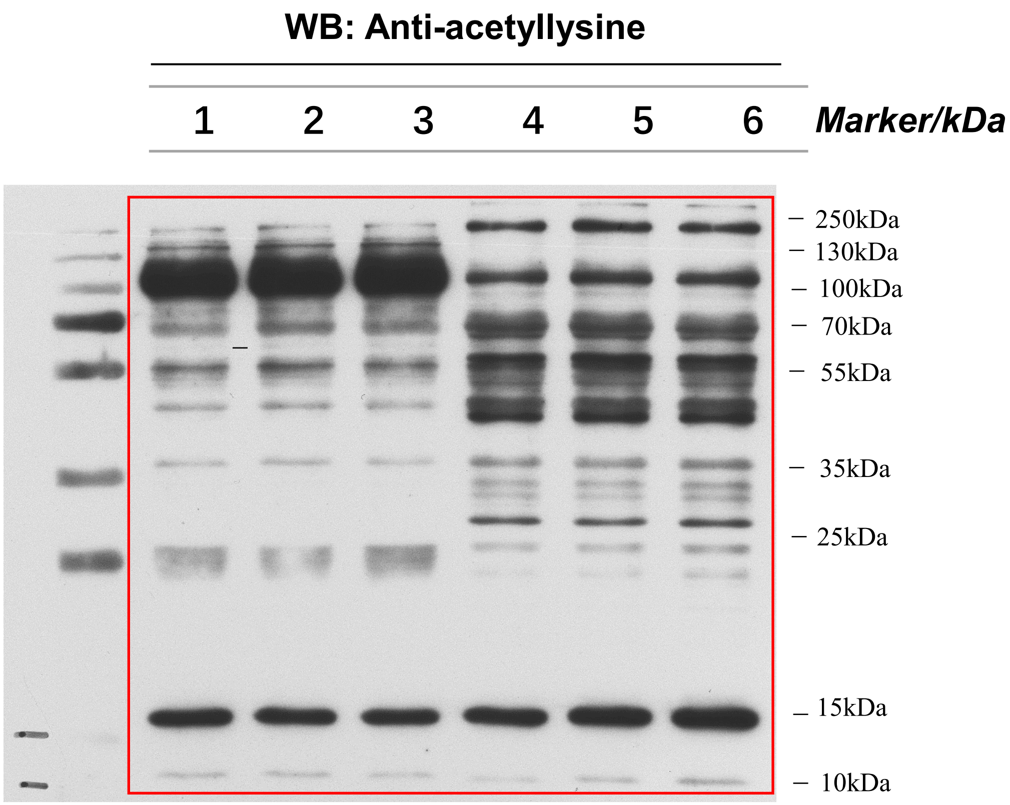

Figure4E

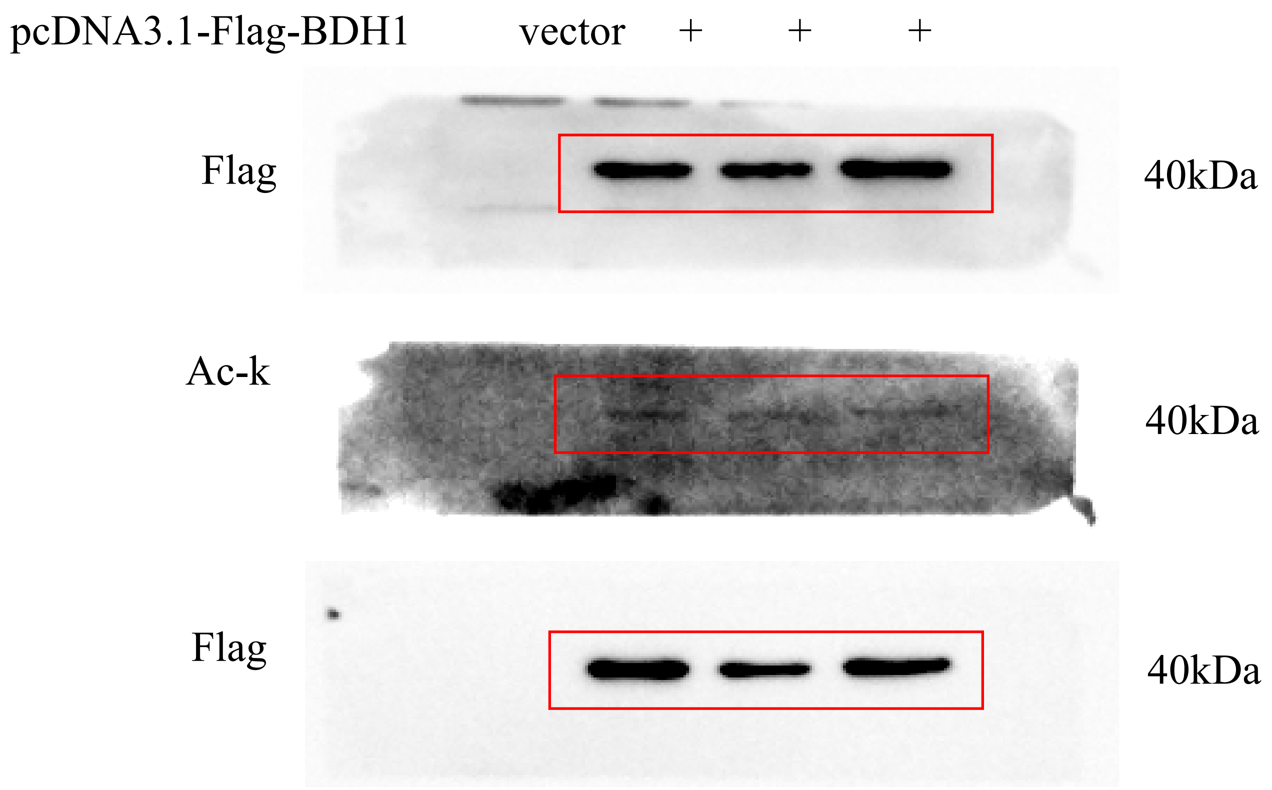

Figure4F

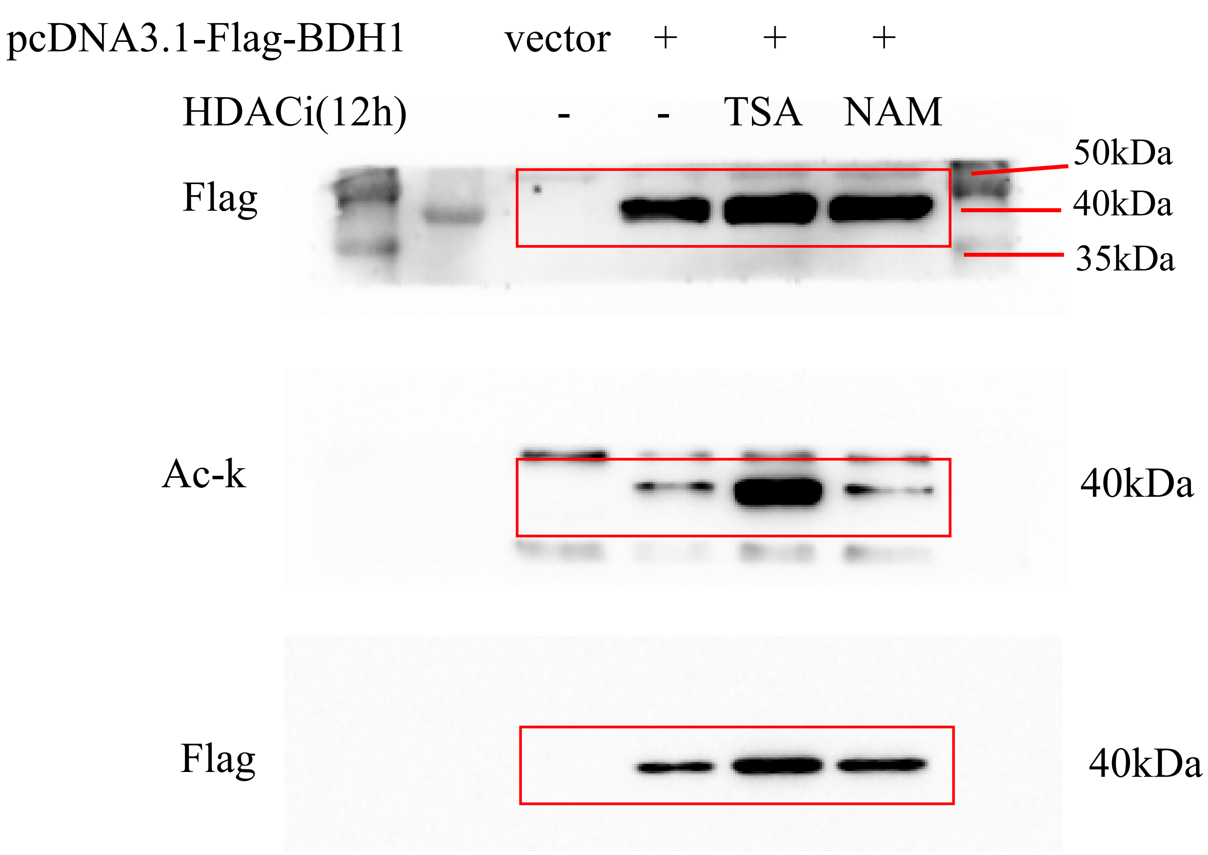
pcDNA3.1-Flag-BDH1 vector + + +
HDACi(12h) - - TSA NAM
Flag
50kDa
40kDa
35kDa
Ac-k
40kDa
Flag
40kDa

Figure5B

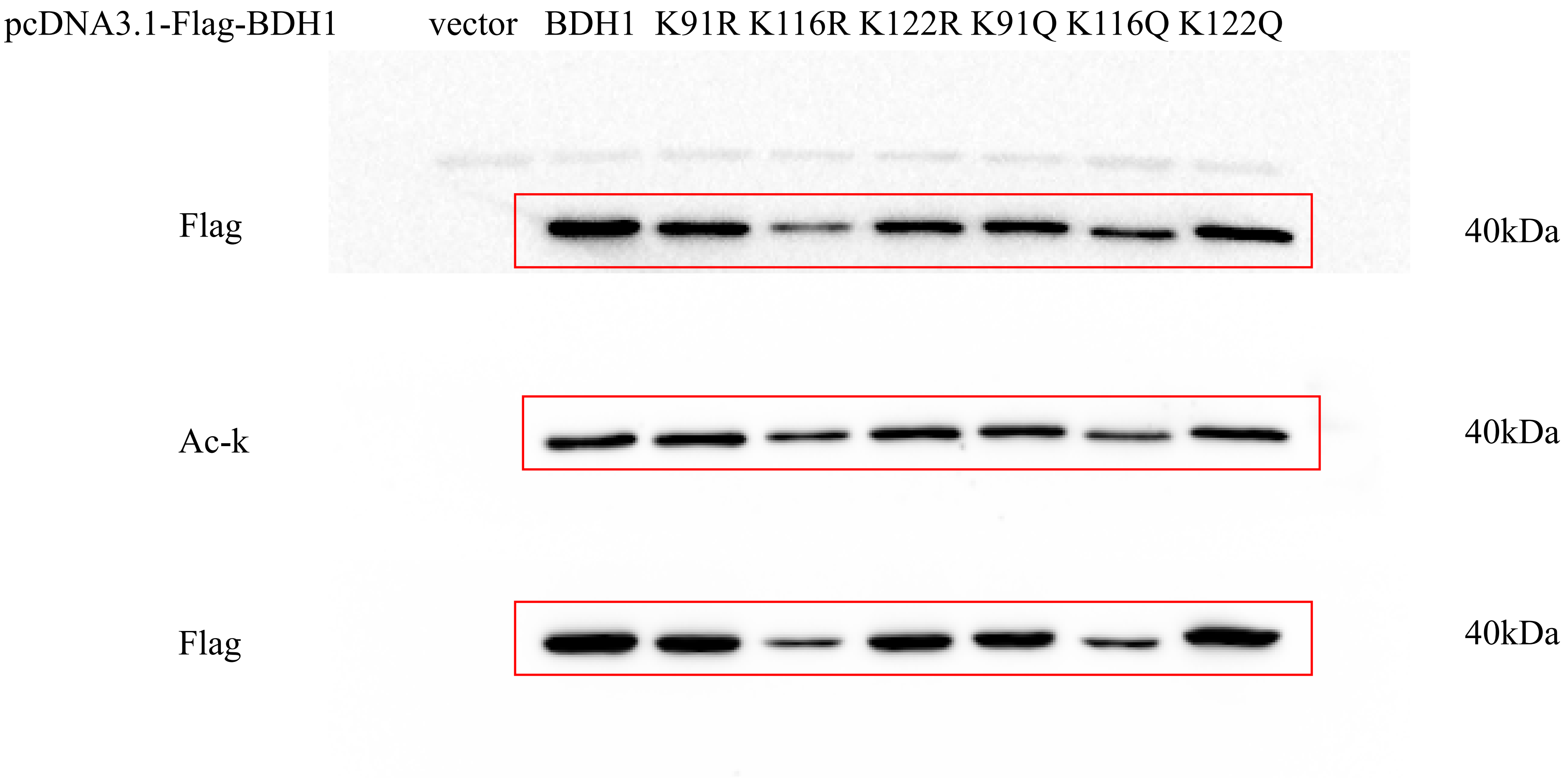
pcDNA3.1-Flag-BDH1
vector
BDH1
K91R
K116R
K122R
K91Q
K116Q
K122Q
Flag
40kDa
Ac-k
40kDa
Flag
40kDa

Figure5C

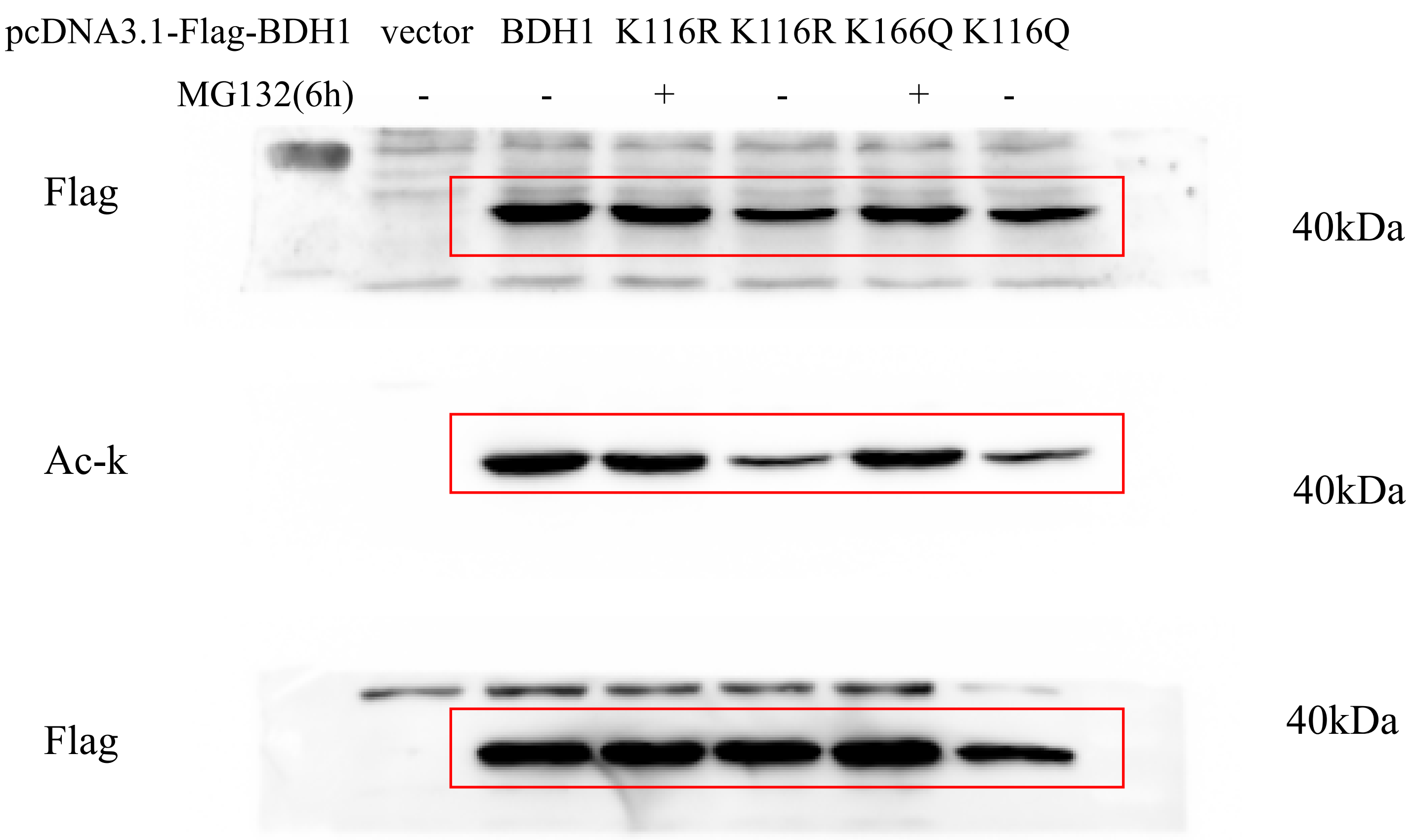
pcDNA3.1-Flag-BDH1 vector BDH1 K116R K116R K166Q K116Q
MG132(6h) - - + - + -
Flag
40kDa
Ac-k
40kDa
Flag
40kDa

Figure5D

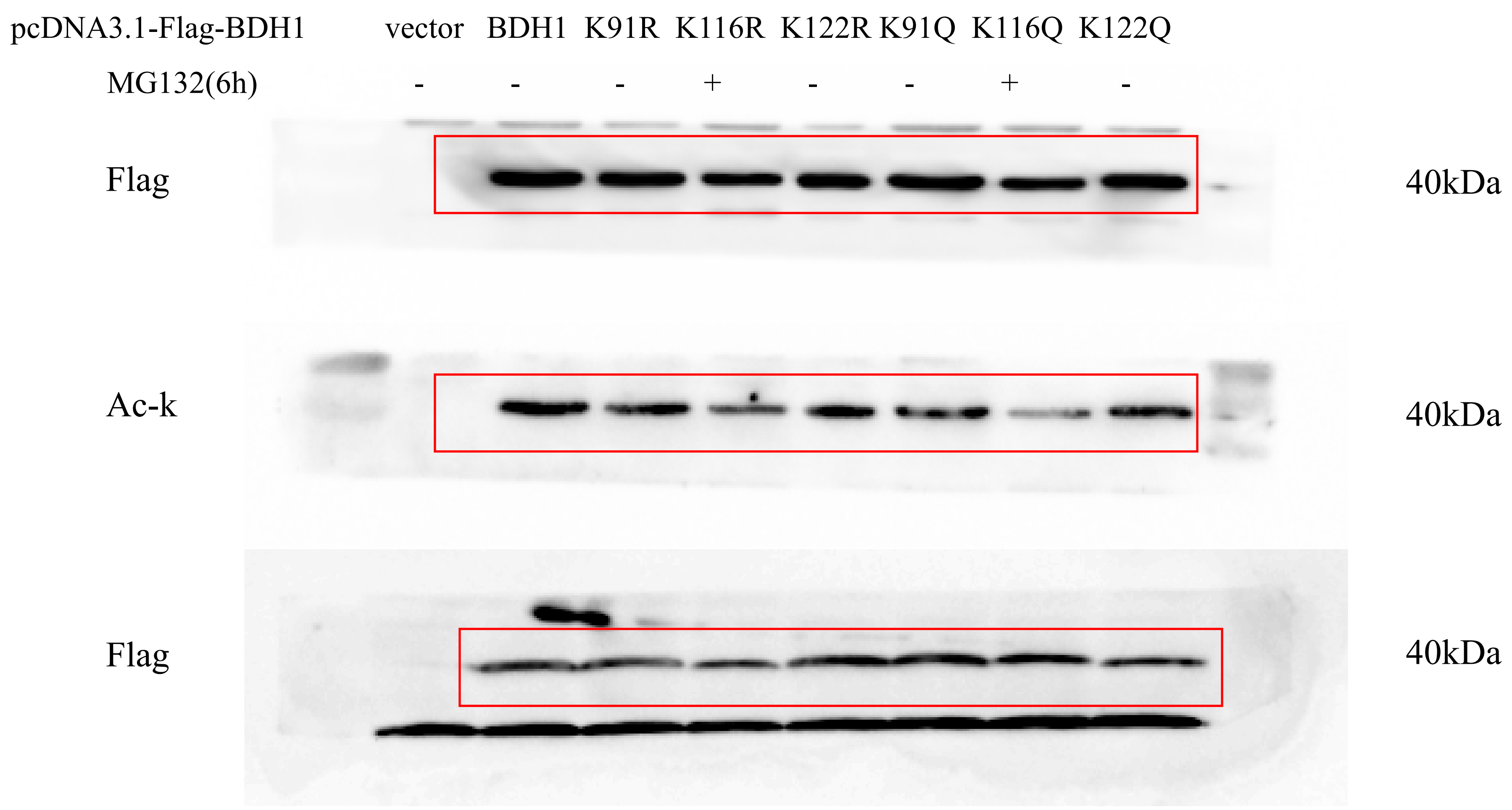
pcDNA3.1-Flag-BDH1
vector BDH1 K91R K116R K122R K91Q K116Q K122Q
MG132(6h)
- - - + - - + -
Flag
Ac-k
Flag
40kDa
40kDa
40kDa
